# Multidisciplinary management of severe odontogenic maxillofacial multi-space infection in a patient with HIV–syphilis coinfection and advanced immunosuppression: a case report

**DOI:** 10.3389/fcimb.2026.1945372

**Published:** 2026-09-03

**Authors:** Jieying Li, Yuanyong Feng, Xueqiang Guo, Wei Shang, Kai Zhou

**Affiliations:** 1 Department of Oral and Maxillofacial Surgery, The Affiliated Hospital of Qingdao University, Qingdao, Shandong, China; 2 School of Stomatology, Qingdao University, Qingdao, Shandong, China; 3 Department of Stomatology, Qingdao Women and Children’s Hospital, Qingdao University, Qingdao, Shandong, China

**Keywords:** human immunodeficiency virus, immune reconstitution, multidisciplinary team, odontogenic maxillofacial space infection, syphilis

## Abstract

**Background:**

HIV–syphilis coinfection poses a substantial challenge in the management of severe odontogenic infections, with few reports addressing maxillofacial multi-space infections in this setting.

**Case presentation:**

A 50-year-old man developed severe left maxillofacial multi-space infection one week after molar extraction, presenting with respiratory distress, trismus, elevated C-reactive protein (235.20 mg/L), computed tomography evidence of gas-forming abscesses, reactive HIV antibody, positive syphilis serology, and a CD4+ T-cell count of 133.76 cells/μL. Emergency tracheostomy and incision and drainage were followed by multidisciplinary management comprising sequential antibacterial therapy, intravenous aqueous penicillin G, early antiretroviral therapy, and trimethoprim–sulfamethoxazole prophylaxis. The patient was decannulated on postoperative day 23 and discharged on day 25; the CD4+ T-cell count rose to 321.53 cells/μL within three weeks. At four-month follow-up, the infection had not recurred, the CD4+ T-cell count reached 415.36 cells/μL, and the RPR had reverted to negative.

**Conclusion:**

In patients with advanced immunosuppression, odontogenic multi-space infection mandates prompt airway control, adequate surgical drainage, sequenced pathogen-specific pharmacotherapy, and multidisciplinary collaboration.

## 1 Introduction

Odontogenic maxillofacial space infections are common acute conditions with potentially life-threatening implications in oral and maxillofacial–head and neck surgery (Shah et al., 2025; Wang et al., 2025). Without timely and effective intervention, these infections can spread rapidly along fascial spaces to involve the submandibular, submental, buccal, parapharyngeal, and pterygomandibular regions, potentially leading to airway compromise, descending necrotizing mediastinitis, sepsis, multiple organ failure, and death (Li et al., 2021). Management is challenging even in immunocompetent hosts; when these infections occur in severely immunosuppressed individuals, the clinical course becomes substantially more perilous. Human immunodeficiency virus (HIV) compromises host immunity and predisposes affected individuals to a wide spectrum of opportunistic infections, often with frequent and occasionally atypical oral manifestations (Coogan et al., 2005). Furthermore, concomitant syphilis may further exacerbate immunosuppression, attenuate typical inflammatory responses, and obscure or mimic other diseases, thereby delaying diagnosis (Scarvaglieri et al., 2025).

HIV and syphilis coinfection represents a substantial public health challenge, particularly given the high prevalence of syphilis among individuals living with HIV (Mauceri et al., 2023; Miao et al., 2024; Arbune et al., 2026). In patients with coinfection, immune function further deteriorates, leading to a progressive decline in CD4+ T-cell counts, an increased frequency of opportunistic infections, and a more atypical course of syphilis (Montenegro-Idrogo et al., 2023; Wu et al., 2023; Miao et al., 2024). In light of the global resurgence of syphilis, including in China (Zhou et al., 2025), clinicians are increasingly likely to encounter such patients in routine practice. In this population, the occurrence of severe multi-space maxillofacial infections necessitates a delicate balance between surgical intervention, empirical anti-infective therapy, the timing of antiretroviral therapy (ART) initiation, and pathogen-specific treatment, while also accounting for potential drug–drug interactions and organ dysfunction (Wu et al., 2023; Arbune et al., 2026). While the treatment principles for each individual condition are well established, published clinical reports detailing this triple clinical scenario remain limited.

Here, we report the diagnostic and therapeutic course of a patient with HIV–syphilis coinfection complicated by severe odontogenic multi-space maxillofacial infection, emphasizing individualized management, timely surgical intervention, and sequenced pharmacotherapy delivered through a multidisciplinary team (MDT) model.

## 2 Case presentation

### 2.1 History and presenting features

A 50-year-old man presented to our emergency department on 1 March 2026 with progressive left facial swelling. His past medical history was unremarkable: he reported no hypertension, diabetes mellitus, cardiac disease, or other chronic systemic conditions, and he was not taking any long-term medications. He had a 30-year smoking history (20 cigarettes per day) and denied alcohol consumption. One week earlier, he had undergone sequential extraction of teeth 24–26 at a private dental clinic for left maxillary posterior pain and had taken only oral anti-inflammatory medication for three days. Progressive swelling, purulent-bloody discharge from the gingival margins, dysphagia, trismus, severe headache, and respiratory distress subsequently developed. On arrival he was acutely ill, wheelchair-dependent, and dysarthric. Physical examination revealed diffuse, tense, tender left maxillofacial and cervical swelling with subcutaneous crepitus; mouth opening was limited to less than one fingerbreadth, and the alveolar bone was exposed at the extraction sockets with abundant purulent discharge.

### 2.2 Laboratory tests

Laboratory tests showed a white blood cell count of 17.93 × 10^9^/L (neutrophils 95.1%, lymphocytes 2.3%), hemoglobin 98 g/L, C-reactive protein (CRP) 235.20 mg/L, and procalcitonin 4.36 μg/L; prothrombin time 16.3 s, fibrinogen 7.85 g/L, and D-dimer 0.88 μg/mL; alanine aminotransferase (ALT) 89 U/L, albumin 29 g/L, and total bilirubin 34.10 μmol/L. Serological screening revealed a positive *Treponema pallidum* antibody (150 S/CO), a positive rapid plasma reagin (RPR) test at a titer of 1:2, and a reactive HIV antibody screening test. Hepatitis B surface (25.95mIU/mL) and core antibodies (3.46IU/mL) were positive with a negative surface antigen (< 0.05PEI/mL); hepatitis C antibody was positive (19.7 S/CO) with a negative antigen, and hepatitis C virus (HCV) RNA was subsequently confirmed to be below the lower limit of detection, a pattern consistent with spontaneous clearance.

### 2.3 Imaging

**Figure 1:**
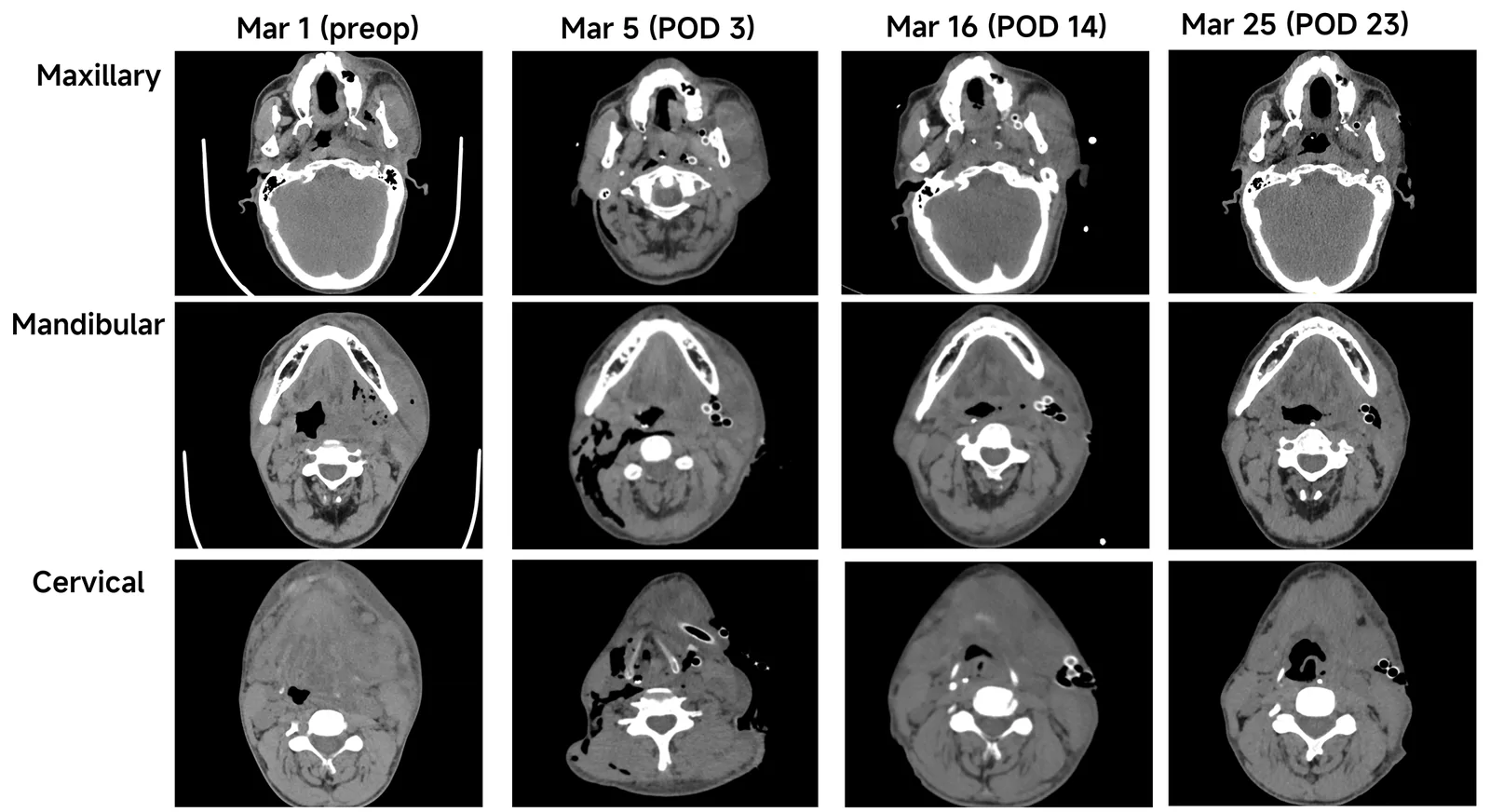
Serial cervical CT at the maxillary (row 1), mandibular (row 2), and cervicothoracic (row 3) levels. Column A (March 1, preop): diffuse left maxillofacial soft-tissue swelling, obliteration of fat planes, and gas-forming multi-space abscesses. Column B (March 5, postoperative day 3): extensive subcutaneous emphysema of the bilateral cervicofacial soft tissues extending to the thoracic inlet. Column C (March 16, postoperative day 14): marked resorption of gas and regression of the space infections. Column D (March 25, postoperative day 23): near-complete resolution with minimal residual gas. preop: preoperative; POD: postoperative day.

Emergency cervical computed tomography (CT) on the night of admission showed diffuse left maxillofacial soft-tissue swelling with obliterated fat planes and gas-forming abscesses extending from the infratemporal region to the submandibular space, with narrowing of the oropharyngeal airway (**Figure 1**, column A). Serial CT scans were obtained during the course: on postoperative day 3 (March 5), extensive subcutaneous emphysema of the bilateral cervicofacial and supraclavicular soft tissues extended to the thoracic inlet (column B); scans during March 9–22 showed gradual gas resorption (a representative scan on March 16 is shown in column C); and on postoperative day 23 (March 25), the infection had almost completely resolved, with minimal residual gas (column D). These serial findings directly informed management: the admission scan prompted emergency drainage, persistent emphysema supported continued broad-spectrum coverage, and stepwise resolution served as an objective prerequisite for de-escalation (**Figure 1**).

### 2.4 Diagnosis and etiological workup

On admission, the patient and his family denied any history of HIV, syphilis, or viral hepatitis. With the family’s consent, samples were sent to the local Center for Disease Control and Prevention (CDC), which confirmed HIV-1 antibody positivity. The absolute CD4+ T-cell count on postoperative day 2 was 133.76 cells/μL, establishing stage 3 HIV infection (AIDS) according to the CDC staging criterion (<200 cells/μL); together with the positive treponemal serology, HIV–syphilis coinfection was diagnosed. After the diagnosis was communicated, the family disclosed that the patient had been registered as HIV-infected at the local CDC in 2008, although no detailed records were available and antiretroviral therapy had never been initiated. HIV viral load measurement was not performed during the acute phase. The hepatitis serology was interpreted as resolved hepatitis B virus (HBV) infection, defined by a negative surface antigen (HBsAg) with positive surface (anti-HBs) and core (anti-HBc) antibodies, and cleared HCV infection, defined by a positive HCV antibody (19.7 S/CO) with a negative HCV antigen and HCV RNA below the lower limit of detection, indicating spontaneous clearance in the absence of any prior antiviral therapy.

Microbiological workup proceeded as follows: culture of drainage fluid obtained intraoperatively on March 2 yielded *Streptococcus anginosus* (aerobic) on March 4; sputum cultures initially showed Gram-positive cocci and Gram-negative bacilli and later normal respiratory flora; fungal G and GM tests and endotoxin testing were negative (March 9); a 13-pathogen respiratory nucleic acid panel detected parainfluenza virus RNA (March 12); and blood cultures showed no aerobic or anaerobic growth (March 16).

### 2.5 Clinical course and management

**Figure 2:**
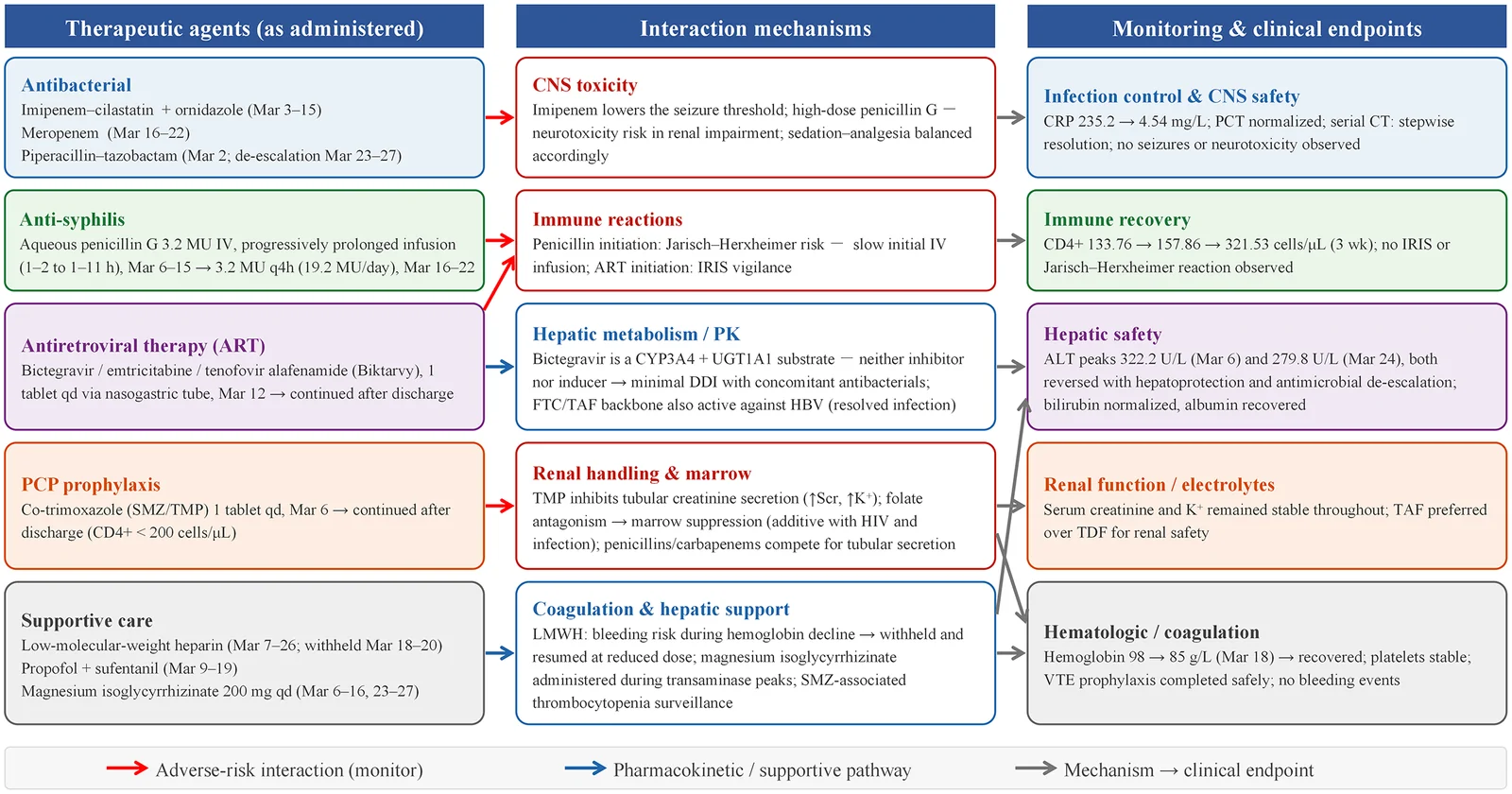
Drug–drug interaction mechanisms and pharmacovigilance strategy during MDT-guided sequenced therapy. Left panel: therapeutic agents as administered (doses and dates); middle panel: principal interaction mechanisms by organ system; right panel: monitoring targets and observed clinical endpoints. Red dashed arrows indicate adverse-risk interactions requiring monitoring; blue solid arrows indicate pharmacokinetic or supportive pathways; gray arrows link mechanisms to clinical endpoints. MDT, multidisciplinary team; HIV, human immunodeficiency virus; CNS, central nervous system; ART, antiretroviral therapy; IRIS, immune reconstitution inflammatory syndrome; JHR, Jarisch–Herxheimer reaction; PCP, *Pneumocystis jirovecii* pneumonia; SMZ/TMP, sulfamethoxazole/trimethoprim; MU, million units; IV, intravenous; qd, once daily; q4h/q6h/q8h, every 4/6/8 hours; NG, nasogastric; PK, pharmacokinetics; DDI, drug–drug interaction; FTC, emtricitabine; TAF, tenofovir alafenamide; TDF, tenofovir disoproxil fumarate; HBV, hepatitis B virus; Scr, serum creatinine; K^+^, serum potassium; LMWH, low-molecular-weight heparin; VTE, venous thromboembolism; CRP, C-reactive protein; PCT, procalcitonin; ALT, alanine aminotransferase; CT, computed tomography; CD4, cluster of differentiation 4; CBC, complete blood count; Hb, hemoglobin; Bil, bilirubin; ALB, albumin.

Because of progressive airway compromise, emergency surgery was performed on the night of admission. Oral intubation was not feasible because of severe trismus; a tracheostomy was therefore performed first, followed by extensive incision and drainage of the left maxillofacial and cervical spaces under general anesthesia. The patient was transferred to the intensive care unit (ICU), and an MDT (Oral and Maxillofacial Surgery, Critical Care Medicine, Infectious Diseases, Clinical Pharmacy, Dermatology, and the affiliated infectious diseases hospital) formulated an individualized, sequenced treatment plan (**Supplementary Table 1** provides the full treatment timeline; **Figure 2**).

**Figure 3:**
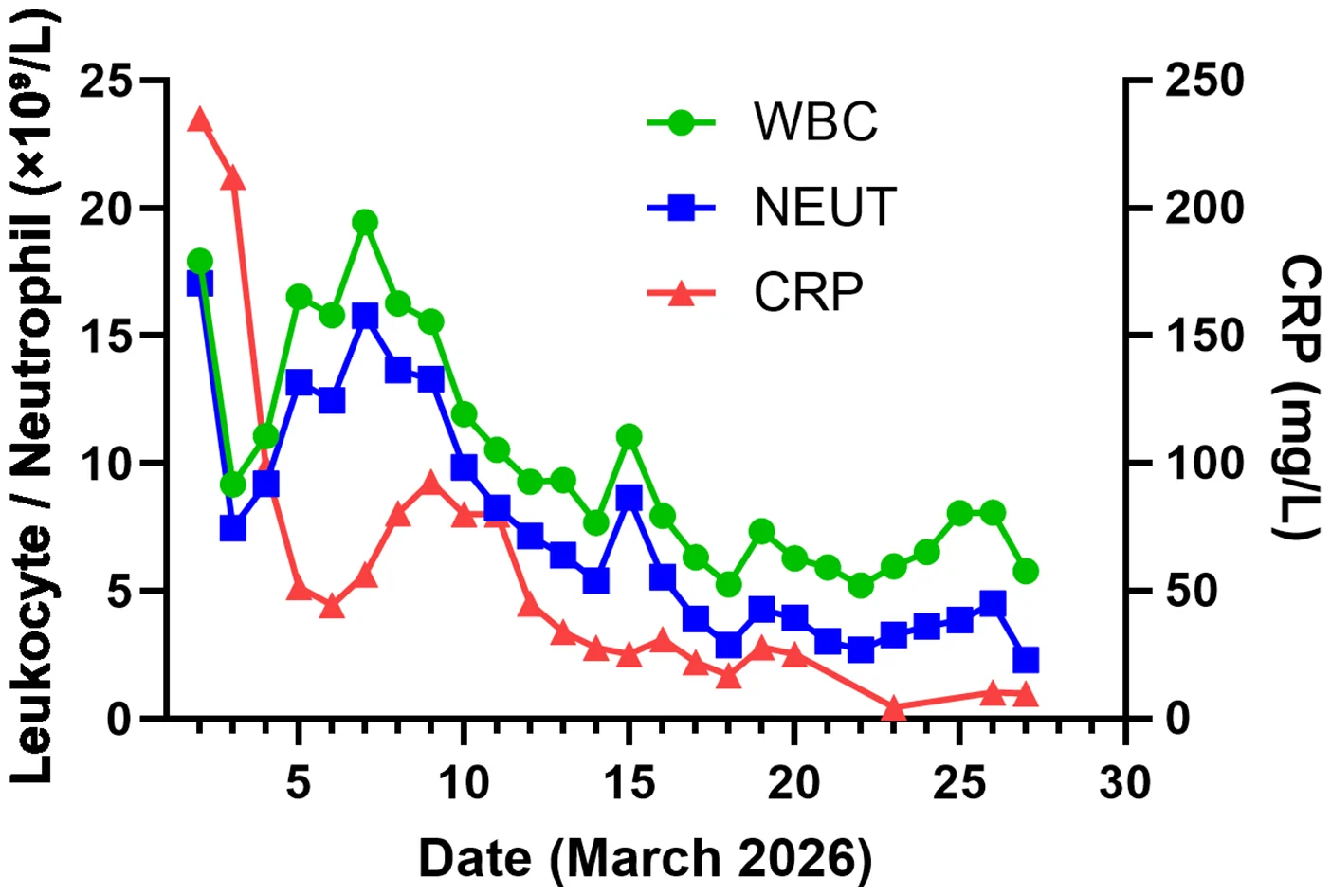
Dynamics of inflammatory markers and infection control. Temporal trends in leukocyte (WBC) and neutrophil (NEUT) counts, and C-reactive protein (CRP) levels during the treatment period. To accommodate the different units and dynamic ranges of these parameters, a dual Y-axis was utilized: the left Y-axis represents leukocyte and neutrophil counts (×10^9^/L, green circles and blue squares, respectively), while the right Y-axis indicates CRP levels (mg/L, red upward-pointing triangles). The data demonstrate an initial peak in inflammatory markers coinciding with the acute phase of infection, followed by a significant and steady decline. By approximately postoperative day 24, all parameters had returned to low, stable levels, indicating effective control of the odontogenic maxillofacial infection.

Antimicrobial therapy: Empirical piperacillin–tazobactam plus ornidazole was initiated before surgery to cover the polymicrobial (streptococcal and anaerobic) spectrum typical of odontogenic multi-space infection. On the following day, given the patient’s critical condition and advanced immunosuppression, the regimen was escalated to imipenem–cilastatin (0.5 g every 6 h) plus ornidazole to extend Gram-negative coverage pending culture results. Subsequently, drainage-fluid culture yielded *Streptococcus anginosus*, which was generally susceptible to penicillin-class β-lactams. Blood cultures and fungal markers remained negative, and no multidrug-resistant pathogen was identified. On hospital day 16, when imipenem–cilastatin became temporarily unavailable, a clinical pharmacy consultation recommended meropenem (1 g every 8 h) as an equivalent within-class substitute. This substitution was additionally favorable because imipenem is more epileptogenic than meropenem, an important consideration given the concurrent escalation of high-dose aqueous penicillin G (Cannon et al., 2014). On hospital day 23, the patient was afebrile, inflammatory markers were declining (**Figure 3**), serial CT showed stepwise resolution (**Figure 1**), and creatinine clearance was 140 mL/min. A further consultation recommended de-escalation to piperacillin–tazobactam (4.5 g every 6 h as a 2-h extended infusion), followed by oral amoxicillin–clavulanate at discharge. Clinical pharmacy consultations were provided repeatedly throughout the course.

Anti-syphilis therapy: The initial dermatology consultation recommended benzathine penicillin G (2.4 million units intramuscularly, weekly for three weeks). In view of the patient’s severe headache and advanced immunosuppression, lumbar puncture was recommended to the family for cerebrospinal fluid examination. However, the family declined further testing, and cerebrospinal fluid (CSF) analysis was therefore not performed. Because neurosyphilis could not be excluded in the setting of advanced immunosuppression, the regimen was changed on hospital day 6 to intravenous aqueous penicillin G (3.2 million units per dose, with the infusion duration progressively prolonged from 1–2 h to 11 h), intensified to 3.2 million units every 4 h (19.2 million units/day) from hospital day 16, with close monitoring for a Jarisch–Herxheimer reaction.

**Figure 4:**
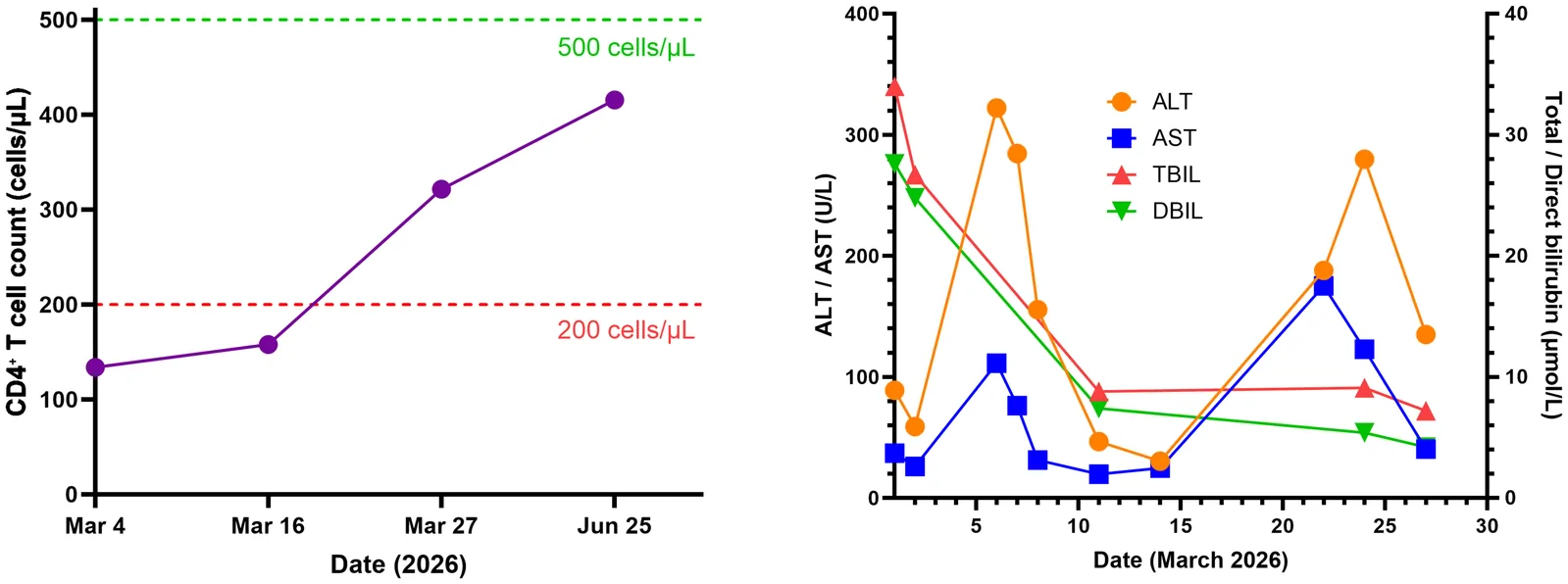
Monitoring of immune reconstitution and hepatic function recovery during therapy and follow-up. **(A)** Temporal trends in the absolute CD4^+^ T-lymphocyte count, shown at the four sampling time points: March 4 (postoperative day 2), March 16 (week 2), and March 27 (week 3) during hospitalization, and June 25, 2026, at outpatient follow-up (note the non-uniform time scale). The red dashed line represents the diagnostic threshold for severe immunodeficiency (200 cells/μL), and the green dashed line indicates the lower limit of the normal range (500 cells/μL). The CD4^+^ T-cell count increased from 133.76 cells/μL at baseline to 415.36 cells/μL at four-month follow-up. **(B)** Hepatic function indices during hospitalization, using a dual Y-axis to accommodate different units and magnitudes: the left Y-axis indicates alanine aminotransferase (ALT) and aspartate aminotransferase (AST) levels (U/L, orange circles and blue squares, respectively), and the right Y-axis indicates total bilirubin (TBIL) and direct bilirubin (DBIL) levels (μmol/L, red and green triangles, respectively). The data illustrate an initial acute liver injury peak around postoperative day 6 and a second transient ALT elevation around postoperative day 24, followed by recovery toward the normal range at discharge.

ART and opportunistic-infection prophylaxis: On hospital day 12 (day 6 after confirmation of the diagnosis), ART with bictegravir/emtricitabine/tenofovir alafenamide (one tablet daily via a nasogastric tube) was initiated. Because the CD4+ T-cell count was below 200 cells/μL (**Figure 4A**), co-trimoxazole (one tablet daily) was administered as prophylaxis against *Pneumocystis jirovecii* pneumonia (PCP).

Supportive care: Magnesium isoglycyrrhizinate (200 mg intravenously once daily) was administered during episodes of transaminase elevation; low-molecular-weight heparin was used for thromboprophylaxis and was temporarily withheld when hemoglobin declined; propofol and sufentanil provided sedation and analgesia during mechanical ventilation; and enteral nutrition was provided via a nasogastric tube. On hospital day 19 the tracheostomy tube was exchanged for a metal cannula and capping trials were begun; on hospital day 20, three additional supraclavicular drains were placed and later removed stepwise.

### 2.6 Outcome and follow-up

Inflammatory markers improved steadily: CRP fell from 235.20 to 4.54 mg/L by hospital day 23, and the white blood cell count normalized (**Figure 3**). Normalization of inflammatory markers (**Figure 3**), together with imaging resolution, guided antimicrobial de-escalation and discharge; hepatic indices (**Figure 4B**) guided hepatoprotective treatment and antimicrobial adjustment. The CD4+ T-cell count increased from 133.76 cells/μL (postoperative day 2) to 157.86 cells/μL (week 2) and 321.53 cells/μL (week 3) (**Figure 4**). Serum ALT peaked at 322.2 U/L (hospital day 6) and again, transiently, at 279.8 U/L (day 24), declining to 135 U/L at discharge; bilirubin normalized and albumin recovered to 33.3 g/L (**Figure 4**). The patient was weaned from mechanical ventilation, decannulated on postoperative day 23, and resumed oral intake. Serial CT confirmed stepwise resolution of the infection (**Figure 1**). He was discharged on postoperative day 25 with oral amoxicillin–clavulanate, co-trimoxazole prophylaxis, and continued ART, and was referred to the infectious diseases hospital for long-term follow-up. Post-discharge follow-up extended to four months. The patient completed the discharge medication course as prescribed and continued bictegravir/emtricitabine/tenofovir alafenamide once daily and co-trimoxazole (one tablet daily) thereafter. The cervicofacial swelling resolved completely, with no recurrence of infection. Repeat testing at a local hospital in late June 2026 showed that the rapid plasma reagin titer had declined from 1:2 at baseline to 1:1 and was reported as negative, indicating an adequate serological response to anti-syphilis therapy; the CD4+ T-cell count reached 415.36 cells/μL on June 25, 2026, exceeding the 200 cells/μL threshold for severe immunodeficiency and approaching the normal range (**Figure 4**).

## 3 Discussion

Severe odontogenic maxillofacial multi-space infection in a patient with HIV-syphilis coinfection and advanced immunosuppression presents a distinctive therapeutic challenge; three treatment-intensive conditions must be managed simultaneously, each influencing the others. Published experience with this triple clinical scenario remains limited; only isolated cases of odontogenic deep neck infection (e.g., Ludwig angina) in HIV-positive patients have been reported (Truong et al., 2023), and none have addressed the additional complexity introduced by active syphilis coinfection. In the present case, emergency airway management, extensive surgical drainage, and sequenced combination pharmacotherapy, coordinated through an MDT, resulted in a favorable outcome.

Oral and maxillofacial infections are prevalent among individuals living with HIV and may manifest as opportunistic infections (Coogan et al., 2005). The fascial spaces within the maxillofacial region are composed of loose connective tissue, which provides minimal resistance to the dissemination of infection. In malnourished and immunosuppressed hosts, a local odontogenic source—such as extraction sockets colonized by oral streptococci like *Streptococcus anginosus*—can escalate to a life-threatening multi-space infection within days (Parhiscar and Har-El, 2001; Parmar et al., 2022). Dental procedures, poor oral hygiene, and immunocompromised states (including AIDS) are recognized predisposing factors for Ludwig angina and deep neck infections (Parmar et al., 2022). In accordance with contemporary series and expert consensus, the management of severe multi-space infections necessitates early airway control, prompt and adequate incision and drainage, and broad-spectrum antimicrobial therapy, alongside intensive care support when systemic involvement is evident (Henry et al., 2021; Li et al., 2021; Perina et al., 2022; Garola et al., 2024; Isankova et al., 2025). Our case exemplifies that these principles remain applicable, albeit more challenging to execute, in the presence of severe immunosuppression: the presenting lymphocyte percentage was merely 2.3%, and the absolute CD4^+^ T-cell count (133.76 cells/μL) confirmed the diagnosis of AIDS. In such patients, peripheral leukocyte counts may not accurately reflect the infection burden; thus, we relied on a combined assessment of CRP, procalcitonin, imaging, and clinical presentation rather than solely on the white blood cell count, and employed serial CD4^+^ T-cell counts to monitor immune recovery.

Antimicrobial management in this case illustrates how broad empirical coverage and stewardship principles can be reconciled in an immunocompromised host. The severity of presentation—sepsis, airway compromise, and a CD4+ T-cell count of 133.76 cells/μL—necessitated broad initial coverage and early carbapenem escalation rather than a conventional stepwise approach. Thereafter, however, therapy was repeatedly reassessed against microbiological and clinical evidence: the isolation of *Streptococcus anginosus*, susceptible to first-line β-lactams, together with negative blood cultures and fungal markers, excluded multidrug-resistant pathogens and defined the point at which narrowing became safe. Each subsequent adjustment had a documented trigger and was made under formal clinical pharmacy consultation: meropenem was substituted when imipenem–cilastatin became unavailable, a change that additionally reduced the additive seizure-threshold risk of concurrent high-dose penicillin G (Cannon et al., 2014); and de-escalation to piperacillin–tazobactam as a 2-h extended infusion was implemented once the patient was afebrile with declining inflammatory markers, resolving imaging findings, and a creatinine clearance of 140 mL/min. Total carbapenem exposure was thus limited to three weeks, and no recurrence occurred during four months of post-discharge follow-up, supporting the feasibility of combining aggressive early coverage with timely de-escalation in this population.

In patients coinfected with HIV and syphilis, immune function progressively declines, and syphilis may exhibit a rapid and atypical course (Wu et al., 2023; Scarvaglieri et al., 2025). Oral syphilis is recognized as a “great imitator” (Smith et al., 2021), and the clinical spectrum of oral secondary syphilis is broader in HIV-infected individuals (Ramírez-Amador et al., 2013). In immunocompromised patients, the management of syphilis necessitates particular caution, as standard treatment regimens may be insufficient. Although benzathine penicillin G (2.4 million units intramuscularly weekly for three doses) is the standard of care for uncomplicated syphilis, its inadequate CSF penetration precludes the achievement of treponemicidal concentrations. In our patient, cerebrospinal fluid examination was recommended in view of his severe headache and advanced immunosuppression but was declined by the family; neurosyphilis could therefore be neither confirmed nor excluded, and this diagnostic uncertainty directly shaped our therapeutic reasoning. Under these circumstances, three considerations supported the empirical escalation to intravenous aqueous penicillin G. First, the patient exhibited a potential neurologic manifestation (severe headache), syphilis of unknown duration, and a CD4^+^ T-cell count of 133.76 cells/μL—well below 350 cells/μL, a recognized risk marker for asymptomatic neurosyphilis in HIV-infected patients (odds ratio [OR] 2.87, 95% confidence interval [CI] 1.18–7.02) (Ghanem et al., 2008). Second, current guidelines recommend CSF examination for HIV-infected patients with syphilis and neurologic symptoms and, when CSF cannot be evaluated, management with a regimen effective against neurosyphilis; under-treatment of occult neurosyphilis—potentially irreversible in an immunocompromised host—was judged a far greater hazard than the incremental risk of intravenous therapy (Workowski et al., 2021). Third, the escalated regimen (3.2 MU every 4 h; 19.2 MU/day) falls within the guideline-recommended neurosyphilis dose of 18–24 MU/day (Workowski et al., 2021), thereby covering both diagnostic scenarios. The subsequent serological response—RPR reversion to negative within three months—supports the adequacy of this approach, although it cannot substitute for CSF evidence. Throughout the entire treatment course, close monitoring was maintained, and no Jarisch-Herxheimer reaction was observed.

The timing and composition of ART are equally pivotal. Current guidelines recommend that patients initiating treatment for an acute opportunistic infection begin ART within two weeks of the diagnosis, provided the patient is clinically stable (Panel on Antiretroviral Guidelines for Adults and Adolescents, 2026). In our case, surgical source control was achieved on the night of admission and broad-spectrum antibacterial coverage was instituted immediately, establishing a clear temporal sequence of “source control first, then ART”. Notably, the patient was antiretroviral-naïve despite reportedly having been registered as HIV-infected since 2008, illustrating how long-standing untreated HIV can remain clinically silent until an intercurrent crisis unmasks advanced immunosuppression. An integrase strand transfer inhibitor (INSTI)–based single-tablet regimen (bictegravir/emtricitabine/tenofovir alafenamide) was selected due to its high barrier to resistance, low potential for drug–drug interactions with concomitant anti-infective agents, and suitability for nasogastric administration. Furthermore, the emtricitabine/tenofovir alafenamide backbone is also effective against HBV, which is relevant given the serological evidence of resolved HBV infection and the associated, albeit low, risk of reactivation. Because the CD4+ T-cell count was below 200 cells/μL, co-trimoxazole prophylaxis against PCP was mandatory and was well tolerated (Panel on Guidelines for the Prevention and Treatment of Opportunistic Infections in Adults and Adolescents with HIV, 2026). The CD4+ T-cell count increased from 133.76 to 321.53 cells/μL within three weeks of treatment initiation, and no immune reconstitution inflammatory syndrome was observed. Immune reconstitution is a major determinant of long-term prognosis in individuals living with HIV, particularly for those who begin treatment with advanced immunosuppression (Li et al., 2026).

Two additional features of this case warrant emphasis. First, transient liver injury occurred twice during the course, with ALT peaks of 322.2 U/L and 279.8 U/L, most plausibly related to the infection itself, carbapenem and high-dose penicillin exposure, as well as subsequent combination therapy. Dynamic monitoring, hepatoprotective treatment with magnesium isoglycyrrhizinate, and timely de-escalation of antimicrobials enabled the completion of all planned pathogen-specific treatments. As summarized in **Figure 2**, this pharmacovigilance framework determined the day-to-day monitoring plan—hepatic, renal and electrolyte, and neurologic surveillance—throughout the treatment course. This underscores the importance of pharmacovigilance when polypharmacy is unavoidable in coinfected patients. Second, the MDT structure—led by Oral and Maxillofacial Surgery and Critical Care Medicine, supported by Infectious Diseases, Clinical Pharmacy, and Dermatology—proved decisive at every critical juncture: airway strategy, antimicrobial escalation and de-escalation, modification of the anti-syphilis regimen, initiation of ART, and prophylaxis against opportunistic infections. Multidisciplinary collaboration has also been demonstrated to improve outcomes in other fulminant maxillofacial and cervical infections, such as descending necrotizing mediastinitis (Zheng et al., 2020).

This report has several limitations. First, as a single-case observation, the findings represent one feasible approach rather than a general recommendation. Second, cerebrospinal fluid examination (declined by the patient’s family) and HIV viral load measurement were not performed during the acute phase; the escalation to intravenous penicillin G therefore remains a risk-averse, guideline-informed empirical decision rather than confirmation of neurosyphilis. Third, the transient liver injury could not be definitively attributed among infection, antimicrobials, and antiretroviral therapy. Fourth, despite favorable four-month follow-up, longer-term data on immune recovery durability and virological suppression are still needed.

## 4 Conclusion

In severely immunosuppressed patients with HIV and syphilis coinfection, odontogenic maxillofacial multi-space infections progress rapidly and atypically. Conventional inflammatory markers may underestimate disease severity; therefore, monitoring CD4+ T-cell counts is essential. Prompt airway control, early and adequate surgical drainage, sequenced anti-infective, anti-syphilis, and antiretroviral pharmacotherapy—while paying attention to drug interactions—along with opportunistic infection prophylaxis and organ support, are key elements of treatment. An MDT approach represents an effective strategy for managing such complex and rare conditions.

## Data Availability

The original contributions presented in the study are included in the article/**Supplementary Material**. Further inquiries can be directed to the corresponding authors.
