# Supplementary material for: Multidisciplinary management of severe odontogenic maxillofacial multi-space infection in a patient with HIV–syphilis coinfection and advanced immunosuppression: a case report
Source: Front Cell Infect Microbiol. 2026 Sep 3;16:1945372. doi: 10.3389/fcimb.2026.1945372 (PMC13581513; doi:10.3389/fcimb.2026.1945372)
Supplement: Supplementary file 1 [file Table1.docx]

Supplementary Material

# 1 Supplementary Figures and Tables

For more information on Supplementary Material and for details on the different file types accepted, please see [here](https://www.frontiersin.org/guidelines/author-guidelines#supplementary-material).

## 1.1 Supplementary Table

| Supplementary Table 1: Timeline of antimicrobial, anti-syphilis, antiretroviral, and supportive treatments. | | | | |
| --- | --- | --- | --- | --- |
| **Category** | **Agent and regimen** | **Route** | **Period**  **(March 2026)** | **Remarks** |
| **Antibacterial** | Piperacillin–tazobactam 4.5 g q6h + ornidazole 0.5 g q12h | IV | Mar 2 | Empirical therapy at surgery |
| **Antibacterial** | Imipenem–cilastatin 0.5 g q6h + ornidazole 0.5 g q12h | IV | Mar 3–15 | Escalation for severe infection; pus culture: *Streptococcus anginosus* |
| **Antibacterial** | Meropenem 1 g q8h | IV | Mar 16–22 | Regimen adjustment |
| **Antibacterial** | Piperacillin–tazobactam 4.5 g q6h (2-h extended infusion) | IV | Mar 23–27 | De-escalation after clinical pharmacy consultation |
| **Antibacterial** | Amoxicillin–clavulanate 500 mg bid | PO | From discharge (Mar 27) | Oral step-down therapy |
| **Anti-syphilis** | Aqueous penicillin G 3.2 MU per dose with progressively prolonged infusion (1–2 to 1–11 h), Mar 6–15 → 3.2 MU q4h (19.2 MU/day), Mar 16–22 | IV | Mar 6–22 | Neurosyphilis could not be excluded; monitoring for Jarisch–Herxheimer reaction |
| **Antiretroviral** | Bictegravir/emtricitabine/tenofovir alafenamide (Biktarvy) 1 tablet qd | NG | Mar 12 → continued | Initiated on day 6 after confirmed HIV diagnosis; FTC/TAF backbone also active against HBV |
| **PCP prophylaxis** | Trimethoprim–sulfamethoxazole 1 tablet qd | PO/NG | Mar 6 → continued | CD4^+^ T-cell count < 200 cells/μL |
| **Hepatoprotection** | Magnesium isoglycyrrhizinate 200 mg qd | IV | Mar 6–27 | During transaminase elevation |
| **Anticoagulation** | Low-molecular-weight heparin | SC | Mar 7–26 (withheld Mar 18–20; reduced dose Mar 21–22) | VTE prophylaxis; withheld during hemoglobin decline |
| **Sedation/analgesia** | Propofol + sufentanil | IV | Mar 9–19 | During mechanical ventilation |
| IV: intravenous; PO: per os; NG: nasogastric; SC: subcutaneous; PCP: Pneumocystis jirovecii pneumonia; VTE: venous thromboembolism; FTC/TAF: emtricitabine/tenofovir alafenamide; | | | | |
